# Use of Natural Antimicrobial Peptides and Bacterial Biopolymers for Cultured Pearl Production

**DOI:** 10.3390/md13063732

**Published:** 2015-06-11

**Authors:** Christelle Simon-Colin, Yannick Gueguen, Evelyne Bachere, Achraf Kouzayha, Denis Saulnier, Nicolas Gayet, Jean Guezennec

**Affiliations:** 1 Ifremer, Centre de Brest, BP 70, 29280 Plouzané, France; E-Mails: Christelle.Simon.Colin@ifremer.fr (C.S.-C.); Nicolas.Gayet@ifremer.fr (N.G.); 2 Ifremer UMR 5244 IHPE, UPVD, CNRS, Université de Montpellier, CC 80, F-34095 Montpellier, France; E-Mails: Yannick.Gueguen@ifremer.fr (Y.G.); Evelyne.Bachere@ifremer.fr (E.B.); 3 Ifremer, UMR 241 EIO, UPF-ILM-IRD, Labex Corail, BP 7004, 98719 Taravao, French Polynesia; E-Mail: Denis.Saulnier@ifremer.fr; 4 Faculty of Sciences, Biochemistry Department, Section III, Lebanese University, Tripoli, Lebanon; E-Mail: achraf_kouzayha@yahoo.fr; 5 AiMB. 17 Rue d’Ouessant, 29280 Plouzané, France

**Keywords:** pearl oyster, exopolysaccharide, tachyplesin, antimicrobial

## Abstract

Cultured pearls are the product of grafting and rearing of *Pinctada margaritifera* pearl oysters in their natural environment. Nucleus rejections and oyster mortality appear to result from bacterial infections or from an inappropriate grafting practice. To reduce the impact of bacterial infections, synthetic antibiotics have been applied during the grafting practice. However, the use of such antibiotics presents a number of problems associated with their incomplete biodegradability, limited efficacy in some cases, and an increased risk of selecting for antimicrobial resistant bacteria. We investigated the application of a marine antimicrobial peptide, tachyplesin, which is present in the Japanese horseshoe crab *Tachypleus tridentatus*, in combination with two marine bacterial exopolymers as alternative treatment agents. In field studies, the combination treatment resulted in a significant reduction in graft failures *vs.* untreated controls. The combination of tachyplesin (73 mg/L) with two bacterial exopolysaccharides (0.5% w/w) acting as filming agents, reduces graft-associated bacterial contamination. The survival data were similar to that reported for antibiotic treatments. These data suggest that non-antibiotic treatments of pearl oysters may provide an effective means of improving oyster survival following grafting procedures.

## 1. Introduction

**Figure 1:**
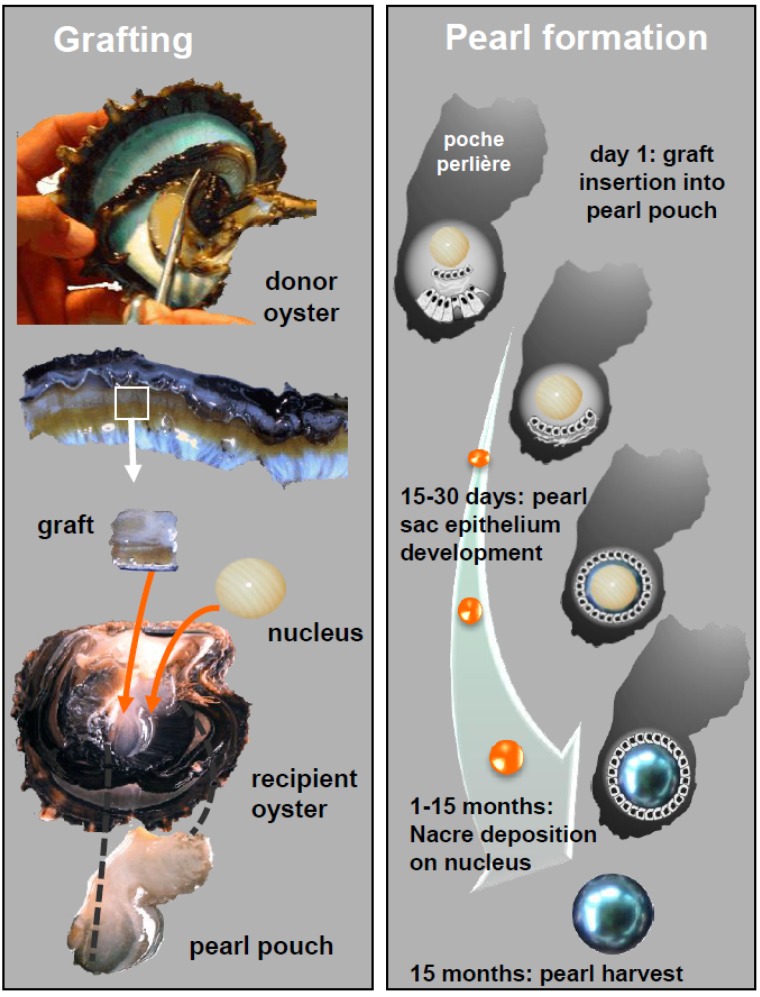
The different steps of the grafting process and pearl formation in the pearl oyster *Pinctada margaritifera*. (illustration C. Montagnani).

The Tahiti pearl farming industry plays a major socio-economic role in French Polynesia. In an increasingly competitive market where the production of high quality pearls becomes essential, research can contribute to secure and ensure a durable production. Cultured pearls are the product of grafting and rearing of *Pinctada margaritifera* pearl oysters in their natural environment. Pearl culture includes several stages. To begin, the pearl oysters are collected and raised to serve either as donor or receiver. The grafting process then takes place following a surgical operation during which the graft, a small piece of mantle tissue, is inserted into the “pearl pocket” of the receiving oyster together with a nacre bead, the nucleus. Once inserted into the receiving oyster, the external epithelial-cells of the graft multiply to form a pearl sac around the nucleus [1]. The pearl sac then starts to deposit calcium carbonate polymorphs layers onto the nucleus. This is the starting point for the future pearl [2]. A rearing period of about 18 months is needed to finally produce a pearl with a sufficiently thick layer of nacre (0.8 mm) (Figure 1).

Unfortunately, nucleus rejections and oyster mortality occur for during the first 45 days following grafting. These phenomena, which typically affect 40% to 50% of oysters within three weeks following the operation, appears to result from bacterial infections or from an inappropriate grafting practice. The development of an inflammatory reaction after insertion of the nucleus and contamination by pathogenic bacteria, combined with the absence of rapid healing of the tissue incised during the graft are the most likely causes of nucleus rejection. Histological anomalies found in *Pinctada margaritifera* oysters could also indicate infection by a pathological agent or be due to grafting practice [3]. To prevent bacterial infections, anesthetics or synthetic antibiotics coated on nuclei have been applied during the grafting practice [4]. Antibiotics and antiseptics have been evaluated as a means of improving hygiene during nuclei insertion operations to reduce post-operative mortality and increase pearl quality [5]. However, the use of such antibiotics such as neomycin and oxytetracycline presents an environmental problem, due to their incomplete biodegradability, and a public health problem because of the potential for development of bacterial resistance.

Considering the tremendous diversity of marine organisms, they are expected to be a source of new products for marine biotechnology including biopolymers and bioactive metabolites. Indeed compounds from marine organisms provide a broad spectrum of natural products exhibiting a wide range of activities which include antimicrobial, antiviral, anticoagulant or antitumor effects [6].

Antimicrobial peptides (AMPs) play a major role in innate immunity, conserved in evolution, and are present in all phyla. They are mostly cationic and amphipathic molecules although they present a great diversity in terms of structural features as well as biological properties and functions. More than 1000 antimicrobial peptides have now been discovered in plants, vertebrates, and invertebrates [7,8,9].

A vast number of marine bacterial extracellular or intracellular biopolymers, such as exopolysaccharides (EPSs) and polyhydroxyalkanoates (PHAs), have been reported over recent decades, and their composition, structure, biosynthesis and functional properties have been extensively studied [10,11,12,13,14,15]. In the course of the discovery of novel biopolymers of biotechnological interest, it is widely accepted that micro-organisms originating from unusual ecosystems will provide a valuable resource not only for exploitation in novel biotechnological processes but also as models for investigating how biomolecules are stabilized when subjected to changing or extreme conditions [16,17,18]. Both biopolymers have been shown to possess either biological and/or filming properties [19,20].

The aim of this study was to evaluate the combination of tachyplesin, a marine antimicrobial peptide (AMP) with two bacterial exopolysaccharides as an alternative route to synthetic antibiotics used for cultured pearls. Experiments were first conducted under laboratory conditions using appropriate biopolymers and AMP then in *in situ* conditions in a pearl oyster farming.

## 2. Results and Discussion

The filming properties of exopolysaccharides depends on many parameters including their chemistry (chemical composition and conformation, branched or linear) and the chemistry of the environment as well. Uronic acids has been demonstrated to encourage adhesion of the polysaccharide onto the surfaces while Ca and Mg ions as present in seawater and oyster induce strong interactions with hydroxyl groups [21,22]. More than 32 exopolysaccharides synthesized by marine bacteria were produced under laboratory conditions for further evaluations. Based on previous information on the chemistry of EPSs and on technical and industrial considerations as well, two EPSs were selected. The chemical composition of the two EPS is listed in Table 1. The first polymer designated as Mo245 was produced by a bacterium isolated in the lagoon of Moorea Island (French Polynesia), belonging to the *Vibrio* genus. During stationary phase growth in batch cultures in the presence of glucose, this bacterium produced a high molecular weight EPS characterized by equal amounts of uronic acid and hexosamine (*N*-acetyl glucosamine and *N*-acetyl galactosamine) along with traces of galactose. EPS designated as GG was provided by G. Geesey from Montana State University (USA). This EPS was mainly composed of neutral sugars as glucose and showed a high polydispersity index (Ip = 4.2) reflecting the heterogeneity of polymer and a relatively low (870 kDa) molecular mass. Low protein content is indicative of a high degree of purity for both exopolymers.

**Table 1:** Chemical composition of the two bacterial EPSs (% total sugars). Nd: non-determined; Gal: Galactose; Glc: Glucose.

Genus	Ref.	Proteins	Neutral Sugars	Uronic Acids	Hexosamines	Substituants
*Vibrio*	Mo 245	<1	2 (Gal)	40	40	Acetate
Nd	GG	<1	90 (Glc)	Tr (<5)	-	-

### 2.1. SEM Analysis

**Figure 2:**
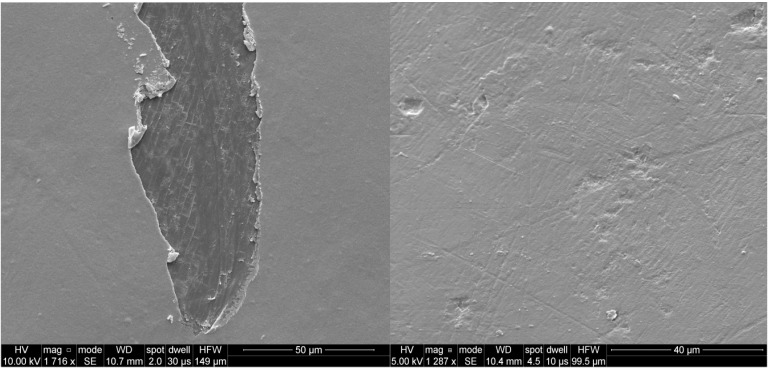
SEM of coated nuclei. Left: EPS/AMP coated nucleus (Scraping indicated the presence of a homogeneous film). Right: Commercial nucleus.

SEM analysis demonstrated the formation of a homogeneous film (0.5 µm to 1 µm thick) on nuclei treated with the two exopolysaccharides and AMP/EPS mixtures. These films were shown to be stable following water rinse cycles. Compared to conventional commercial nuclei, filming with these biopolymers significantly reduced surface asperities with a smoothing effect (Figure 2).

### 2.2. Biopolymer Antibacterial Activity

The antibacterial activity reported for some bacterial strains has been proposed to be due to either polyanionic macromolecules including proteins and exopolysaccharides suggested to inhibit bacterial respiration [23,24] or cell-bound brominated compounds [25,26,27]. While several authors reported antimicrobial effects against various bacteria for EPSs [28,29], we did not find any evidence of an antimicrobial activity or of cytotoxicity for any of the all exopolysaccharides considered in the present study. Similar results were found for both marine and non-marine microbial strains tested.

### 2.3. Laboratory Experiments

With the aim to investigate the existence of bacterial pathogens associated with post graft mortalities, a total of 204 bacterial colonies exhibiting various colors, shapes and size were isolated during grafting operations of the cultivated pearl oysters *P. margaritifera*. Ninety-nine bacteria have been isolated from graft and 105 from pearl pouch. Phenotypic analyses revealed that Gram negative bacteria represent 75% of the isolated strains and *Vibrio* are the predominant bacteria. In samples collected from pearl pouch several days after the graft, *Vibrio* represents 63% of the Gram negative bacteria.

**Figure 3:**
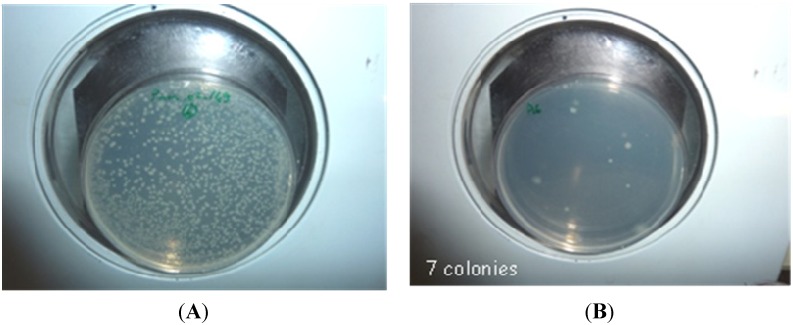
Bacterial growth of a suspension of *Vibrio* (pMAR-02-149) after incubation in the presence of (**A**) uncoated nuclei; (**B**) coated nuclei EPS Mo 245 and tachyplesin.

In that four experimental conditions (A to D) were tested under laboratory conditions including biopolymers alone (A and C) and tachyplesin associated with EPSs (B and D). After 3 months of storage, all coated nuclei B and D (EPS + tachyplesin) still inhibited *Vibrio* growth. Coating with EPS preserved the antimicrobial activity of the peptide (Figure 3).

### 2.4. In Situ Experiments

**Figure 4:**
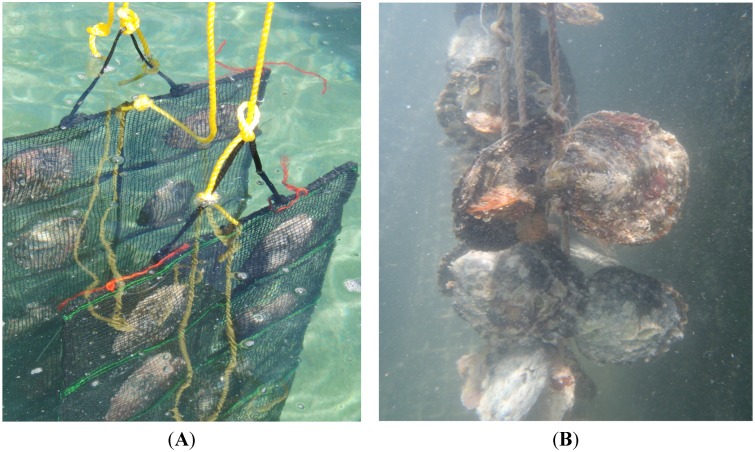
Pearl oysters culture after grating. (**A**) During the first 40 days after the graft, pearl oysters are cultured in retention baskets to assess the rejection of nucleus; (**B**) The shells are then drilled on the side and pearl oysters are tied with a nylon thread to a cord.

**Figure 5:**
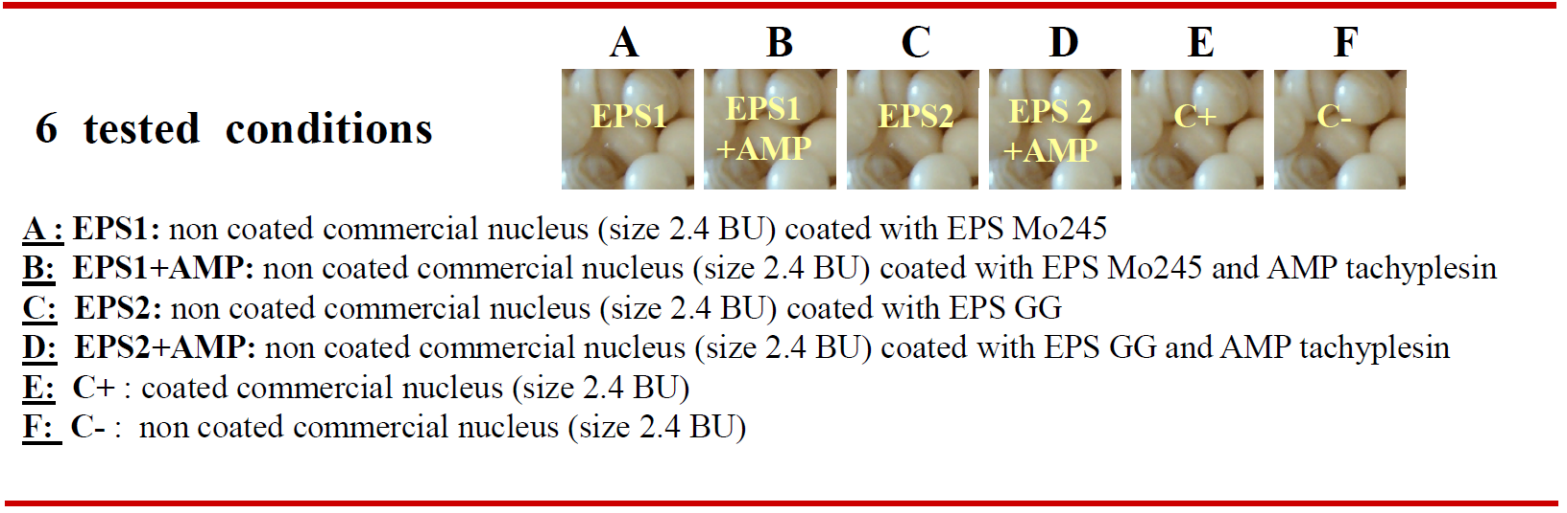

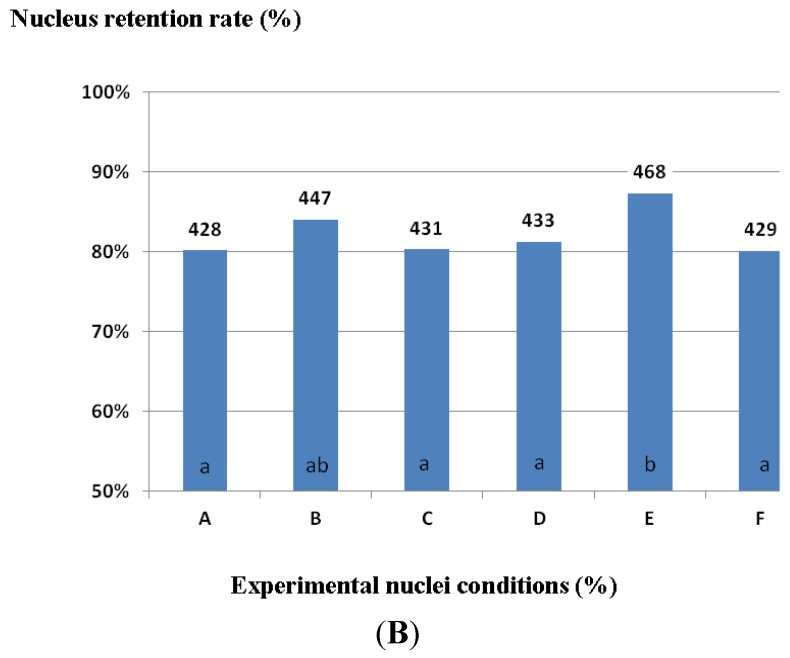
Evaluation of the nuclei retention rate depending on its coating (**A**) Experimental grafting protocol used to evaluate EPS and EPS/PAM experimental nuclei (A to D conditions) with its controls, commercial nuclei (E) and non-coated nuclei (F). Only one nucleus size was used (2.4 BU). Each donor oyster contributed equally to the 6 tested conditions (A to F); (**B**) Nucleus retention rate of grafted pearl oysters (40 days post-graft) depending on the nucleus used. The number of pearl oysters is indicated above each histogram. Statistically homogeneous group are shown by a letter (a, b) inside the histograms using a Chi-2 test at significance level of 0.05.

Approximatively, 500 grafts were performed for each nuclei condition. Nucleus retention and oyster mortality rates were evaluated after 40 days and 15 months of immersion under natural conditions (Figure 4). After 40 days no significant differences were observed within the four different experimental conditions (A, B, C and D). However, the EPS Mo245 associated with the tachyplesin (B) showed a better retention rate than the non coated nucleus (F) and as good as the commercial one (E) (Figure 5A,B). Additionally, coating with tachyplesin-EPSs solution induced a lesser oyster mortality compared to non-treated samples and a similar mortality to commercialized antibiotic treated nuclei.

**Figure 6:**
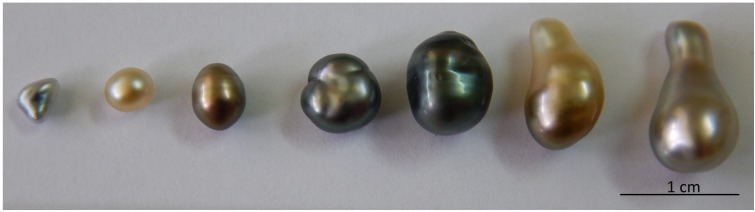
Keshi pearls produced by the pearl oyster *Pinctada margaritifera*.

At the end of the experiments (To +15 months), and compared to commercial nuclei (E), nuclei treated with an EPS/AMP mixture showed a significant increase of pearls of high commercial value (assigned mainly to the proportion of round to semi-round high grade pearls). Furthermore, a decrease in the proportion of keshis was observed comparing EPS/AMP nuclei to non-coated nuclei (F). Keshi pearls correspond to non-nucleated pearls typically formed as by-products of pearl cultivation and referred to those pearls formed when a bead nucleus was rejected. Because they have no nucleus, keshi pearls are composed entirely of *P. margaritifera* nacre (Figure 6). Identical results were observed with nuclei coated with the mixture EPS-tachyplesin and commercialized ones. This is interesting because the results obtained with an environmentally friendly coating are as good as with commercial nucleus. Another advantage to using exopolysaccharide associated with AMP is their binding capability. Bacterial EPSs contain ionizable functional groups such as carboxyl, amine, sulfate and to a lesser extent hydroxyl groups that enable these biopolymers to bind metals [30,31]. Chelation of calcium and magnesium by these exopolysaccharides is in favor of the formation of an homogeneous nacre at the end of the biomineralization process [32].

## 3. Material and Methods

### 3.1. Microbial Biopolymers

Marine bacteria associated with unusual physical and chemical microenvironments have demonstrated their ability to produce unusual extracellular polymers in an aerobic carbohydrate-based medium including exopolysaccharides (EPSs) and polyhydroxyalkanoates (PHAs). Biopolymer production and subsequent chemical analyses of bacterial EPSs are described in previous works [33,34]. Briefly the global composition (neutral sugars, uronic acids, and hexosamine) of the different EPSs were determined using colorimetric methods, while monosaccharide composition and ratios were determined using GC and GC/MS analyses. Non carbohydrate substituants were characterized by HPLC and/or NMR analyses.

### 3.2. Antimicrobial Peptide

Many AMPs have already been isolated from different marine organisms [35]. Tachyplesin is an antimicrobial peptide extracted from hemocytes of the Japanese horseshoe crab *Tachypleus tridentatus* [36]. This AMP (KWCFRVCYRGICYRRCD) displays a potent activity against pathogenic marine Vibrios such as the ones present in pearl oysters [37,38]. Tachyplesin was produced by chemical synthesis by the subcontractant GeneCust (http://www.genecust.com/fr/). The synthetic peptide was analyzed by mass spectrometry and antimicrobial activity assay.

### 3.3. Nuclei

Nuclei were purchased from “Perles Nucleus Science Tahiti”, PNST (BP40146, Fare Tony, Papeete, Tahiti, French Polynesia).

### 3.4. Coated Nuclei Preparation

Nuclei were placed in an aqueous solution of exopolysaccharides (0.5% w/w) for a few hours under gentle agitation. The resulting coated nuclei were simply dried on an absorbent paper for 2 h at room temperature. The filming properties of biopolymers were then evaluated by Scanning Electron Microscopy (SEM).

EPS coated nuclei were then incubated for 48 h at 4 °C into an aqueous solution of tachyplesin at 73 mg/L (32 µM) corresponding to 10 times the MIC (Minimum inhibitory concentrations) as determined for strain *Vibrio* Pmar-02-149 involved in bacterial infections in *Pinctada margaritifera*. The resulting coated nuclei were dried for 24 h.

### 3.5. Scanning Electron Microscopy

For scanning electron microscopy (SEM), dried filmed nucleus was mounted on scanning electron microscopy sample stub and coated in a vacuum with 150 Å of gold (SCD040, Balzers Union, Liechtenstein). Samples were observed using an FEI Quanta 200 scanning electron microscope.

### 3.6. Antimicrobial Activity and Cytotoxicity of the Molecules

Antimicrobial activity of exopolysaccharides (EPS) was assayed against several bacteria including the Gram-positive *Micrococcus luteus* CIP 53.45 and *Bacillus subtilis* ATCC 6633, and Gram-negative *Escherichia coli* SBS 363, *E. coli* ATCC 8739, *Staphylococcus aureus* ATCC 6538 and *Pseudomonas aeruginosa* ATCC 9027 along with marine surface pioneering bacteria (*Flavobacterium* spp., *Vibrio natriegens*, *Alteromonas macleodii*, *Pseudomonas* spp. and *Pseudoalteromonas* spp.). Moreover, several marine *Vibrio* spp. isolated from pearl oyster *Pinctada margaritifera* (*Vibrio tubiashi* EL2, *Vibrio harveyi Takapoto* 177, *Vibrio* SP1 Pmar-02-149) along with the fungal pathogen *Candida albicans* ATCC 10231 were also used to evaluate the activity spectrum. Minimal inhibitory concentrations (MIC) were investigated in duplicate by the liquid growth inhibition assay based on the procedure previously described [39]. EPS cellular toxicity was evaluated by a hemolytic assay as described by Niidome *et al.* (2000) [40] using sheep blood cells (BioMérieux, France).

### 3.7. Antimicrobial Property of Coated Nuclei

Prior to experiments conducted under natural conditions, the antimicrobial property of the tachyplesin was evaluated in the presence of EPSs acting as filming agents. The direct grafting of tachyplesin on nuclei was also compared to the association of the antimicrobial peptide and biopolymers. The antimicrobial activity of coated nuclei was analyzed as previously described by liquid growth inhibition assay. Coated or uncoated nuclei were incubated in a suspension of *Vibrio* SP1 Pmar in Poor Broth supplemented with NaCl 0.5 M. After under gentle agitation during 18 h at 20 °C, the bacterial suspensions were transferred on microplates. Growth was monitored spectrophotometrically at 620 nm on a Multiscan microplate reader. Besides, serial dilutions of the bacterial suspension were plated onto Zobell agar plates and colonies were counted after 24 h incubation at 20 °C.

### 3.8. In Situ Experiments

*In situ* experiments were performed at the Gauguin’s Pearl farm on the atoll of Rangiroa in French Polynesia in June 2011. Up to 3050 nuclei were treated with the two EPSs or associated with the tachyplesin and compared to non-treated samples and nuclei covered with an antibiotic containing synthetic polymer (commercial nuclei, PNST society, Tahiti). The conventional coating protocol of the PNST society was used. Briefly 1 kg of nuclei (approximately 2000 nuclei size 2.4 BU, a Japanese measure which equals to approximately 7.2 mm diameter) were placed in a rotating drum (2 rev/min) and the EPS solution (5 g/L) was injected in 5 times of 10 mL (total 50 mL) for 90 min at temperature room. The nuclei were then placed under vacuum for final drying for 2 to 3 h at room temperature and further stored at 4 °C under vacuum bag free of air. Resulting nuclei are referenced as A and C for EPS Mo 245 and GG, respectively.

For the grafting of tachyplesin, 1000 nuclei A and C were incubated in an aqueous solution of tachyplesin at 73 mg/L (32 µM). For this purpose, the tachyplesin solution (73 mg/L) was injected into the rotating drum following the same procedure as that used for EPS. The resulting EPS/AMP nuclei were recovered, dried under vacuum, and preserved at 4 °C in sealed bags. These were designated as nuclei B and D for EPS Mo 245 + tachyplesin and EPS GG + tachyplesin, respectively.

All coated nuclei were compared to either commercial nuclei coated with an antibiotics containing synthetic polymer (positive control, designated as nuclei E) or non-treated samples nuclei (negative control designated as nuclei F).

## 4. Conclusions

The use of antibiotics combined with petrochemical derived polymer covering the nucleus used in pearl culture could be detrimental for the environment. A marine peptide, the tachyplesin, associated with two bacterial exopolysaccharides acting as filming agents, gave similar performances to antibiotics in terms of both oyster mortality and nucleus rejection rates. Bacterial exopolysaccharides formed a homogeneous and stable coating on the nucleus while the presence of incorporated tachyplesin ensured the desired antimicrobial property. The use of these environmental friendly coatings combined with a marine peptide could be considered as a promising green alternative to prevent costly bacterial infections in pearl farming industry. In terms of both an environmental and economical point of view, tachyplesin associated with either EPSs gave satisfactory data for professionals compared to commercial treatments. Optimization studies are in progress to improve both the coating protocol and the tachyplesin/EPS ratio with the aim of obtaining high grade quality pearls. In a similar way, additional experiments are conducted with Polyhydroxyalkanoates (PHAs) [41]. PHAs are non-water soluble polymers and form a stable film on the surface of the nucleus. Interesting data were observed after 30 days of exposure, in that such polymers are expected to preserve the antimicrobial activity of the tachyplesin. Long term experiments are in progress to evaluate PHAs as filming agents associated with AMP.
